# Risk Evaluation of the NCD Risk Calculator for Open Pancreaticoduodenectomy in Elderly Patients: A Validation Study

**DOI:** 10.1002/ags3.70045

**Published:** 2025-05-28

**Authors:** Nana Kimura, Ayaka Itoh, Ayano Sakai, Katsuhisa Hirano, Kenta Yagi, Naoya Takeda, Kazuto Shibuya, Isaku Yoshioka, Kenta Murotani, Tsutomu Fujii

**Affiliations:** ^1^ Department of Surgery and Science, Faculty of Medicine, Academic Assembly University of Toyama Toyama Japan; ^2^ School of Medical Technology Kurume University Kurume Japan; ^3^ Biostatistics Center Kurume University Kurume Japan

**Keywords:** mortality, NCD risk calculator, pancreaticoduodenectomy, poor postoperative course, postoperative complication

## Abstract

**Background:**

There is no clear indication for surgery in pancreaticoduodenectomy (PD) for the elderly patients. The aim of this study was to use real‐world data to investigate the usefulness of preoperative risk assessment with the risk calculator available in the National Clinical Database (NCD) in Japan.

**Methods:**

A retrospective analysis of 311 patients aged ≥ 65 years who underwent PD was performed. In addition to background factors, preoperative predicted incidence rates calculated with the risk calculator, as well as visceral fat analysis items, were analyzed. Patients with (1) serious postoperative complications, (2) a decline in postoperative activities of daily living (ADL), or (3) discharge to a place other than home were defined as having a poor postoperative course. All patients were randomly assigned to the training cohort (*n* = 209) or validation cohort (*n* = 102).

**Results:**

Comparisons of patient characteristics revealed no differences between the training and validation cohorts. In the training cohort, multivariate analysis revealed that “Predicted incidence of postoperative ADL decline” of ≥ 44.8% (OR 4.68; *p* = 0.031) and “Predicted incidence of Clavien–Dindo grade IV or higher” of ≥ 9.2% (OR 5.92; *p* = 0.025) among those calculated with the risk calculator were independent predictors of a poor postoperative course. Among patients with 2, 1, and none of these factors, 100%, 47.4%, and 15.7%, respectively, had a poor postoperative course.

**Conclusion:**

A “Predicted incidence of postoperative ADL decline” and “Predicted incidence of Clavien–Dindo grade IV or higher” in the NCD risk calculator were useful predictors of a poor postoperative course after PD.

## Graphical abstract

This study investigated the utility of the Japanese NCD risk calculator for preoperative assessment in elderly patients undergoing pancreaticoduodenectomy (PD), using a validation study design. In the training cohort, two preoperative factors—the predicted incidence of postoperative ADL decline ≥ 44.8% and Clavien–Dindo grade IV or higher complications ≥ 9.2%—were identified as independent predictors of a poor postoperative course, and these findings were validated in a separate cohort. These results suggest that the NCD risk calculator is a valuable tool for stratifying surgical risk in elderly PD patients.

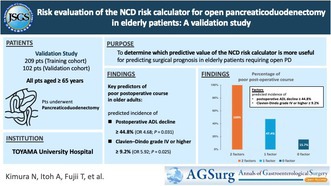

## 1 Introduction

Pancreaticoduodenectomy (PD) is a highly invasive procedure that may cause considerable postoperative complications, such as pancreatic fistula, biliary fistula, postoperative bleeding, infections, and even multiorgan failure, as well as significant postoperative decline in activities of daily living (ADL) [1]. The complication rate for PD is reported to be 40%–60%, and the mortality rate is 2%–5%, the highest among abdominal surgeries [2, 3, 4]. Although the number of minimally invasive PD cases, such as those involving robotic procedures, has increased in recent years, more than half of all PD cases are still treated with open abdominal surgery. The proportion of elderly individuals in the global population is increasing, and there is an increasing need to consider the indications for PD in elderly patients. Elderly patients typically exhibit considerably reduced functional and nutritional capacity, more frequent cognitive and mental health problems, and significantly poorer tolerance of cancer treatments. These aspects translate into longer recovery periods, increased complications, and higher expected mortality among elderly patients after highly invasive procedures such as PD. Therefore, the surgical indications must be carefully determined.

Previous studies from several countries have considered the safety and efficacy of PD in elderly patients, but definitive conclusions are lacking [1, 5, 6]. There are no specific indications as to how to select patients from among the elderly patient candidates for whom surgery is indicated, and at what age the criteria would be applied. There are also a few reports addressing this topic. In clinical practice, the indication for surgery in elderly individuals is determined based on a comprehensive assessment of multiple factors, including performance status, cognitive function, independent living ability, and the presence of severe comorbidities, in the absence of universally established criteria. An appropriate preoperative evaluation will lead to safer management; therefore, it is necessary to identify the preoperative factors that contribute to the surgical prognosis in elderly patients treated with PD.

In the current study, we used a risk calculator from the National Clinical Data (NCD) to evaluate the perioperative risk of open PD. The risk calculator is an NCD feedback tool that can calculate predictive values such as mortality and complication rates for patients undergoing surgery by means of risk models (8 main gastroenterological surgeries, including PD) constructed on the basis of registration data. However, since many predictors are calculated from the NCD risk calculator, there are no criteria for which predictors should be emphasized and what their cutoff values are. The purpose of the current study was to determine which predictive value of the NCD risk calculator is more useful for predicting surgical prognosis in elderly patients requiring open PD with internal validation procedures. The development of clear indicators for the determination of surgical indications can contribute to safer surgical indications and perioperative management.

## 2 Methods

### 2.1 Patients and Study Design

**FIGURE 1:**
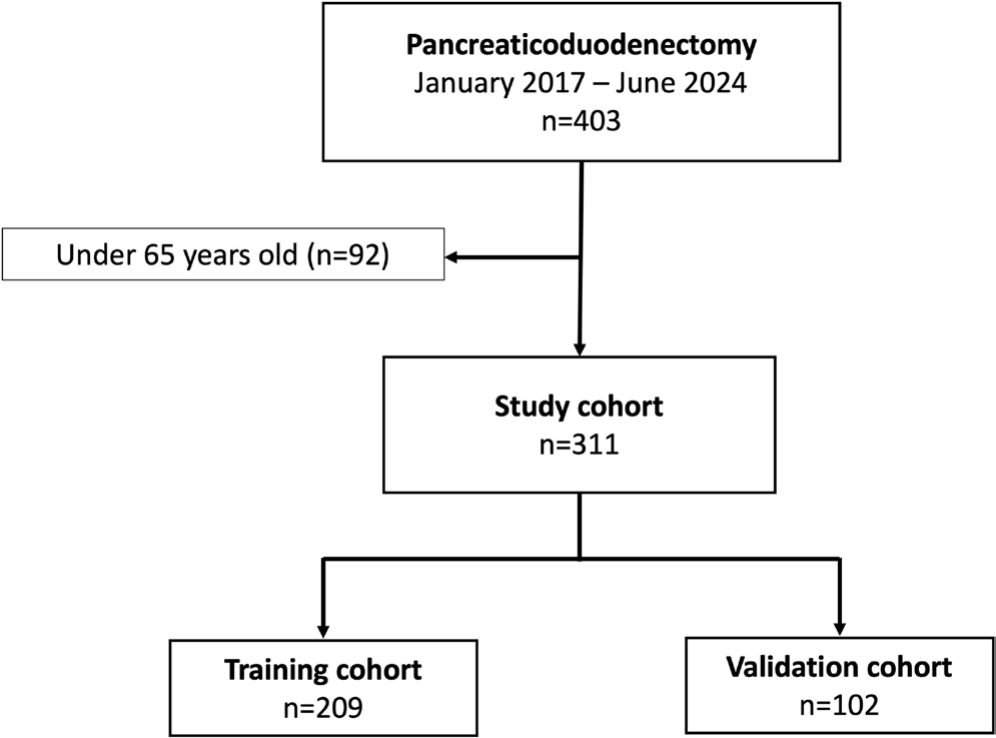
Patient flow diagram.

A prospectively maintained pancreatic resection database at Toyama University Hospital (Toyama, Japan) was queried to identify 578 patients who underwent pancreatectomy between January 2017 and June 2024. Distal and total pancreatectomy, middle‐segment preserving pancreatectomy, minimally invasive pancreatectomy, such as laparoscopic or robotic procedures, and other pancreatic resection procedures were excluded [7]. Among the 403 patients with open PD, 311 were 65 years of age or older at the time of surgery and were selected because the NCD risk calculator performs all risk calculations only for those 65 years of age or older. The patients were randomly divided into two cohorts: the training cohort (*n* = 209) and the validation cohort (*n* = 102). The patient flow diagram is summarized in Figure 1.

Patients were defined as having a poor postoperative course if they had a serious postoperative complication (Clavien–Dindo Grade IV or V), and/or were discharged to a facility other than home, and/or if there was worsening per the Barthel index compared with the preoperative state.

The reasons for using these three items in the definition of poor postoperative outcomes are respectively as follows:

Barthel Index: A decline in the Barthel Index after surgery indicates reduced daily activity performance due to surgical stress, complications, or prolonged hospitalization. See the separate section for additional details.

Discharge to a Facility Other Than Home: This measure reflects the need for further rehabilitation or long‐term care, suggesting suboptimal recovery.

Serious Postoperative Complications (Clavien‐Dindo Grade IV or V): Grade IV or V complications are life‐threatening, often requiring intensive care unit management, and significantly affect both short‐term and long‐term outcomes.

### 2.2 Ethical Statement

The study was reviewed and approved (Ref. No. R2023022) by the Ethics Committee of the University of Toyama and complied with the Strengthening the Reporting of Observational Studies in Epidemiology (STROBE) guidelines [8]. The study conformed to the provisions of the Declaration of Helsinki. Informed consent was obtained from all the participants through an opt‐out form. The study received ethical approval for the anonymization of patient data, the absence of risks to the patient, and the potential benefit for adequate postoperative management on the basis of unbiased information.

### 2.3 Surgical Treatment

Open subtotal gastrectomy with lymphadenectomy (SSPPD) was the standard of care for tumors of the pancreatic head, distal common bile duct, or peripancreatic tumors. Reconstruction was performed according to the modified Child method with Roux‐en‐Y reconstruction. Pancreatojejunostomy was performed via modified Blumgart anastomosis [9]. Gastrointestinal reconstruction was performed by an automated stapling device or by an antecolic route with hand‐sewn anastomosis. Braun anastomosis was performed in all cases.

### 2.4 NCD Risk Calculator

In 2011, the Japanese NCD launched an annual web‐based registration system that collects data on 1.6 million surgical procedures from over 4000 hospitals. Through the NCD feedback website (https://registry3.ncd.or.jp/karte/page/feedback/index), preoperative patient information—such as basic demographics, emergency status, ADL (1 month and immediately before surgery), laboratory results, performance status, tumor type, planned operative procedures, and so on—is entered into the NCD risk calculator. Once submitted, the system automatically calculates predicted 30‐day survival, surgery‐related mortality, the incidence of complications (Clavien–Dindo grade IV or higher), and Grade C pancreatic fistula. For patients aged 65 and older, additional outcomes including delirium, falls, ADL decline, and discharge to facilities other than home are also provided. Detailed variable definitions are available on the NCD website (http://www.ncd.or.jp/).

### 2.5 The American College of Surgeons‐National Surgical Quality Improvement Program (ACS NSQIP) Surgical Risk Calculator

The ACS NSQIP surgical risk calculator is a widely used tool that was originally developed in 2013, and data collected from 393 hospitals between 2009 and 2012 are used in the calculation. It enables an estimation of the risk of 8 postoperative complications using 21 preoperative factors (spanning the procedure, demographics, and comorbidities) [10]. This risk calculation provides estimated risks and predicted lengths of stay for 12 different postoperative complications. The ACS NSQIP calculator was accessed online at http://riskcalculator.facs.org/.

### 2.6 Barthel Index

The Barthel index is a tool introduced by Mahoney and Barthel in 1965 to assess functional status and the ability to perform daily life activities in patients after a stroke [11]. In this study, we used a 10‐item scale, which included the following parts: feeding, walking, transfers, climbing stairs, grooming, dressing, bathing, toilet use, bowels and bladder, and bathing. The score ranges from 0 (total dependence) to 100 (complete independence). Each item can be scored as 0, 5, 10, or 15 points [11]. In the current study, we used the Barthel index, with scores assigned as follows: severe dependence (0–50), moderate dependence (51–75), and mild dependence/independence (76–100).

### 2.7 Body Composition Assessment

In this study, all patients underwent a preoperative abdominal computed tomography (CT) scan to assess the extent of disease. SYNAPSE VINCENT by Fujifilm was used to measure the visceral fat area and psoas muscle volume. Manual tracing of the CT scan at the three lumbar levels was used to measure the cross‐sectional areas of the right and left psoas muscles. The psoas muscle index was calculated by normalizing the cross‐sectional area of the psoas muscle to the square of the patient's height (cm^2^/m^2^). Psoas muscle density (PMD) was the mean CT value of the left and right psoas major cross sections at the L3 level.

### 2.8 Data Collection

We collected patient data, including preoperative factors such as age, sex, body mass index, American Society of Anesthesiologists‐physical status, mobility aids, dementia, Barthel index, ADL, Charlson comorbidity index, NCD risk calculator, ACS NSQIP surgical risk calculator, and body composition assessment of visceral fat and muscle from preoperative CT images. The perioperative factors included surgical procedures, operative time, blood loss volume, blood transfusion, vascular resection, other organ resection, and incidence of postoperative complications according to the Clavien–Dindo classification [12], length of hospital stay, and mortality.

### 2.9 Internal Validation

In this study, a random split method was used to split the dataset. The entire dataset (*n* = 311) was used, with 65% used in the training cohort and the remaining 35% used in the validation cohort. The training and validation cohorts were split randomly, and stratified sampling was applied to ensure that the distribution of each class was kept equal in both cohorts.

The training cohort included 209 patients, and the validation cohort included 109 patients. The training cohort was used to develop the model, and the validation cohort was used to assess the performance of the model. The performance of the model was assessed by means of the area under the receiver operating characteristic (ROC) curve (AUC).

### 2.10 Statistical Analysis

A biostatistician (K.M.) was responsible for the statistical analysis. Binomial and continuous variables were compared by means of the Pearson chi‐square test and nonparametric Mann–Whitney *U* test, respectively. All optimal cutoff values for the logistic regression to predict poor postoperative course were determined using the Youden index. We used univariate and multivariate logistic regression to generate odds ratios, including 95% confidence intervals, for clinical factors that would predict a poor postoperative course. The variables included in the multivariate models for poor postoperative course had a *p* value of 0.05 or less in the univariate analysis. No statistical hypothesis was set, and multiplicity was not accounted for because all analyses were performed in an exploratory manner. A *p* value < 0.05 was considered statistically significant. Statistical analyses were performed via JMP Pro Version 17 (SAS Institute Inc.).

## 3 Results

### 3.1 Characteristics and Preoperative Status of the Training Cohort and the Validation Cohort

Data on the characteristics and preoperative status of the training cohort and the validation cohort are shown in Table 1. In the comparison of the training cohort (*n* = 209) and the validation cohort (*n* = 102), the median ages were 74 and 73 years, respectively. The male/female ratio was 128/81 versus 60/42, the median BMI was 21.5 versus 22.1, and there were no significant differences.

**TABLE 1:** Comparison of clinical characteristics of training and validation cohorts.

	Training cohort (*n* = 209)	Validation cohort (*n* = 102)	*p*
Age (years)^a^	74 (65–89)	73 (65–86)	0.658
Sex (male/female)	128/81	60/42	0.682
Body mass index (kg/m^2^)^a^	21.5 (15.1–40.8)	22.1 (15.7–33.2)	0.466
Walking assistive device (yes/no)	18/191	11/91	0.536
ASA‐PS^a^	2 (1–4)	2 (2–4)	0.111
Smoking history (yes/no)	97/112	40/62	0.230
Brinkmann index^a^	27.5 (0–2640)	0 (0–1600)	0.307
Oral anticoagulant medication (yes/no)	21/188	6/96	0.221
Oral antiplatelet medication (yes/no)	24/185	10/92	0.283
Steroid medication (yes/no)	4/205	7/95	0.027
Mortality within 30 days after surgery	2/208	0/101	0.006
Comorbidity			
Cerebrovascular disease (yes/no)	23/186	7/95	0.245
Cardiovascular diseases (yes/no)	17/192	13/89	0.196
Respiratory diseases (yes/no)	27/182	13/89	0.966
Metabolic and endocrine diseases (yes/no)	154/55	68/34	0.827
Collagen diseases (yes/no)	7/202	9/93	0.040
Renal diseases (yes/no)	18/191	11/91	0.536
Gastrointestinal diseases (yes/no)	104/105	58/44	0.118
Dementia (yes/no)	2/207	1/101	0.984
Charlson comorbidity index^a^	5 (3–1)	5 (3–10)	0.219
Perioperative clinical characteristics			
Primary disease (pancreatic cancer/cholangiocarcinoma/IPMN/NEN/Other)	83/64/43/7/12	47/31/15/4/5	0.837
Tumor characteristics (malignant/benign)	117/32	91/11	0.278
Neoadjuvant chemotherapy (yes/no)	69/140	40/62	0.282
Preoperative biliary drainage (yes/no)	97/112	56/46	0.860
Combined resection of the portal vein (yes/no)	58/151	29/73	0.900
Combined resection of the major artery (yes/no)	7/202	3/99	0.848
Combined resection of other organs (yes/no)	15/194	5/97	0.443
Operative time^a^	558 (249–1036)	564 (287–1034)	0.881
Intraoperative blood loss^a^	620 (80–4600)	625 (5–4160)	0.912
Intraoperative blood transfusion (yes/no)	30/179	9/93	0.295
Postoperative hospital stay (day)^a^	25 (9–112)	25 (10–212)	0.864
NCD risk calculator			
Predicted incidence of surgery‐related mortality (%)^a^	1.1 (0.4–20.6)	1.1 (0.4–5.7)	0.489
Predicted incidence of surgery‐related mortality (%)^a^	2.3 (1.0–27.8)	2.6 (1.0–7.6)	0.075
Predicted incidence of Clavien‐Dindo grade IV or higher (%)^a^	4.0 (1.5–54.2)	4.2 (1.5–17.9)	0.119
Predicted incidence of pancreatic fistula grade C (%)^a^	3.9 (1.5–49.1)	4.5 (1.5–17.4)	0.389
Predicted incidence of postoperative delirium (%)^a^	7.8 (4.1–47.6)	6.9 (4.1–36.2)	0.603
Predicted incidence of fall risk (%)^a^	4.2 (0.9–91.6)	4.0 (0.9–76.7)	0.903
Predicted incidence of physical function decline at 30 days postoperatively (%)^a^	22.4 (17.9–88.8)	21.8 (17.9–85.7)	0.721
Predicted incidence of postoperative ADL decline compared to preoperative (%)^a^	3.5 (0.8–98.2)	3.1 (0.8–99.1)	0.509
Predicted incidence of discharge to a place other than home (%)^a^	4.2 (1.3–80.9)	4.2 (1.3–85.9)	0.992
ACS NSQIP surgical risk calculator			
Serious complication (%)^a^	24.5 (15.0–41.2)	24.1 (16.0–37.8)	0.992
Readmission (%)^a^	16.0 (10.5–24.6)	15.5 (12.0–24.0)	0.711
Death (%)^a^	2.0 (0.5–8.0)	2.0 (0.5–9.7)	0.709
Discharge to Nursing or Rehab facility (%)^a^	11.0 (2.7–43.0)	19.0 (3.0–38.0)	0.525
Body composition assessment			
Preoperative visceral fat percentage (%)^a^	28.9 (4.4–54.4)	30.1 (3.9–57.1)	0.612
Preoperative visceral fat (cm^3^)^a^	2068.1 (190.7–6689.9)	2241.5 (189.1–8213.7)	0.595
Preoperative subcutaneous fat (cm^3^)^a^	2327.0 (241.5–7461.1)	2417.4 (22.2–6803.0)	0.432
Preoperative psoas muscle density^a^	41.6 (12.6–53.7)	42.8 (19.9–56.9)	0.238

Abbreviations: ACS NSQIP, The American College of Surgeons‐National Surgical Quality Improvement Program; ADL, activities of daily living; ASA‐PS, American Society of Anesthesiologists‐physical status; IPMN, intraductal papillary mucinous neoplasm; NCD, National Clinical Database; NEN, neuroendocrine neoplasm.

^a^ Values are medians (ranges).

There were no significant differences between the two cohorts when the comorbidities of gastrointestinal, cardiovascular, and cerebrovascular diseases were compared. In the validation cohort, preoperative medication with steroids was significantly more common. There were no differences in surgery‐related factors, such as operative time, intraoperative blood loss, or surgical procedure.

The predicted risk calculated with the NCD risk calculator was not different between the two cohorts. Furthermore, the predicted risk calculated with the ACS NSQIP surgical risk calculator also revealed no differences between the two cohorts. No differences were found in the body composition assessment calculated with SYNAPSE VINCENT.

### 3.2 Factors Predicting a Poor Postoperative Course

The factors predicting poor postoperative course in all PD patients over 65 years of age are shown in Tables 2 and 3.

**TABLE 2:** Univariate analysis of risk factors predicting a poor postoperative course in the training cohort.

		*n*	Univariate		
			OR	95% CI	*p*
Age	78 ≤	68	4.54	2.21–9.30	< 0.001
	< 78	141			
Sex	Male	128	0.99	0.49–1.99	0.969
	Female	81			
Body mass index (kg/m^2^)	23.8 ≤	51	1.17	0.54–2.55	0.687
	< 23.8	158			
ASA‐PS	1,2	131	0.36	0.17–0.78	0.009
	3,4	50			
Smoking history	Yes	97	1.13	0.57–2.25	0.720
	No	112			
Oral anticoagulant medication	Yes	21	1.32	0.45–3.84	0.611
	No	188			
Oral antiplatelet medication	Yes	24	3.55	1.45–8.72	0.006
	No	185			
Steroid medication	Yes	4	1.38	0.14–13.57	0.785
	No	205			
Comorbidity					
Cerebrovascular disease	Yes	23	4.77	1.93–11.80	< 0.001
	No	186			
Cardiovascular diseases	Yes	17	1.29	0.40–4.19	0.672
	No	192			
Respiratory diseases	Yes	27	1.20	0.45–3.20	0.715
	No	182			
Metabolic and endocrine diseases	Yes	112	1.45	0.72–2.92	0.292
	No	97			
Collagen diseases	Yes	7	0.68	0.08–5.67	0.720
	No	202			
Renal diseases	Yes	18	0.82	0.22–2.92	0.742
	No	191			
Gastrointestinal diseases	Yes	97	0.61	0.30–1.22	0.162
	No	112			
Dementia	Yes	2	1.72	0.90–3.11	0.991
	No	207			
Charlson comorbidity index	< 6	89	1.37	0.69–2.71	0.372
	6 ≤	120			
Perioperative clinical characteristics					
Primary disease	Pancreatic cancer	83	0.96	0.48–1.94	0.920
	Other	126			
Tumor characteristics	Malignant	177	4.24	0.97–18.53	0.055
	Benign	32			
Neoadjuvant chemotherapy	Yes	69	1.98	0.88–4.41	0.097
	No	140			
Preoperative biliary drainage	Yes	97	0.99	0.50–1.98	0.992
	No	112			
Combined resection of the portal vein	Yes	58	1.10	0.52–2.33	0.807
	No	151			
Combined resection of the major artery	Yes	7	0.68	0.08–5.77	0.720
	No	202			
Combined resection of other organs	Yes	15	3.03	1.01–9.06	0.048
	No	194			
Operative time	< 832	11	5.59	1.61–19.45	0.007
	832 ≤	198			
Intraoperative blood loss	< 790	76	1.68	0.84–3.36	0.141
	790 ≤	133			
Intraoperative blood transfusion	Yes	30	1.61	0.66–3.93	0.297
	No	179			
Postoperative hospital stays (day)	< 27	92	2.34	1.17–4.74	0.016
	27 ≤	117			
NCD risk calculator					
Predicted incidence of death at 30 days postoperatively (%)	1.0 ≤	114	2.36	1.13–5.93	0.023
	< 1.0	95			
Predicted incidence of surgery‐related mortality (%)	2.1 ≤	123	43.58	1.56–8.20	0.003
	< 2.1	86			
Predicted incidence of Clavien–Dindo grade IV or higher (%)	9.2 ≤	19	7.33	2.72–19.75	< 0.001
	< 9.2	190			
Predicted incidence of pancreatic fistula grade C (%)	5.8 ≤	51	1.84	0.87–3.85	0.108
	< 5.8	158			
Predicted incidence of postoperative delirium (%)	8.0 ≤	99	2.94	1.42–6.08	0.004
	< 8.0	110			
Predicted incidence of fall risk (%)	4.3 ≤	104	4.74	2.13–10.55	< 0.001
	< 4.3	105			
Predicted incidence of physical function decline at 30 days postoperatively (%)	24.3 ≤	76	2.16	1.08–4.31	0.029
	< 24.3	133			
Predicted incidence of postoperative ADL decline compared to preoperative (%)	44.8 ≤	16	86.49	2.25–18.64	< 0.001
	< 44.8	193			
Predicted incidence of discharge to a place other than home (%)	4.2 ≤	135	3.97	1.58–9.95	0.003
	< 4.2	74			
ACS NSQIP surgical risk calculator					
Serious complication (%)	25.6 ≤	88	2.60	1.29–5.25	0.007
	< 25.6	121			
Readmission (%)	16.0 ≤	107	2.79	1.33–5.83	0.007
	< 16.0	102			
Death (%)	1.9 ≤	120	7.20	2.69–19.25	< 0.001
	< 1.9	89			
Discharge to nursing or rehab facility (%)	14.0 ≤	81	6.26	2.91–13.45	< 0.001
	< 14.0	128			
Body composition assessment					
Preoperative visceral fat percentage (%)	49.9 ≤	6	4.34	0.84–22.36	0.079
	< 49.9	203			
Preoperative visceral fat (cm^3^)	5580 ≤	7	5.95	1.28–27.70	0.023
	< 5580	202			
Preoperative subcutaneous fat (cm^3^)	4636.9 ≤	19	2.04	0.73–5.75	0.176
	< 4636.9	190			
Preoperative psoas muscle density	38.25 ≤	143	0.40	0.20–0.80	0.010
	< 38.25	66			

Abbreviations: ACS NSQIP, The American College of Surgeons‐National Surgical Quality Improvement Program; ADL, activities of daily living; ASA‐PS, American Society of Anesthesiologists‐physical status; CI, confidence interval; NCD, National Clinical Database; OR, odds ratio.

**TABLE 3:** Multivariate analysis of risk factors predicting a poor postoperative course in the training cohort.

		*n*	Multivariate		
			OR	95% CI	*p*
Age	78 ≤	68	1.06	0.31–3.50	0.928
	< 78	141			
ASA‐PS	1,2	131	0.63	0.24–1.65	0.346
	3,4	50			
Oral antiplatelet medication	Yes	24	1.89	0.32–5.91	0.458
	No	185			
Comorbid cerebrovascular disease	Yes	23	2.36	0.66–6.12	0.224
	No	186			
Combined resection of other organs	Yes	15	3.12	0.14–9.43	0.677
	No	194			
Operative time	< 832	11	0.78	0.39–3.27	0.382
	832 ≤	198			
Postoperative hospital stays (day)	< 27	92	0.58	0.59–4.28	0.470
	27 ≤	117			
NCD risk calculator					
Predicted incidence of death at 30 days postoperatively (%)	1.0 ≤	114	0.56	0.17–1.87	0.344
	< 1.0	95			
Predicted incidence of surgery‐related mortality (%)	2.1 ≤	123	2.03	0.61–6.77	0.252
	< 2.1	86			
Predicted incidence of Clavien‐Dindo grade IV or higher (%)	9.2 ≤	19	5.92	1.25–28.00	0.025
	< 9.2	190			
Predicted incidence of postoperative delirium (%)	8.0 ≤	99	0.62	0.17–2.26	0.473
	< 8.0	120			
Predicted incidence of fall risk (%)	4.3 ≤	104	2.12	0.51–8.82	0.304
	< 4.3	105			
Predicted incidence of physical function decline at 30 days postoperatively (%)	24.3 ≤	76	0.52	0.18–1.56	0.246
	< 24.3	133			
Predicted incidence of postoperative ADL decline compared to preoperative	44.8 ≤	16	4.68	1.15–19.08	0.031
	< 44.8	193			
Predicted incidence of discharge to a place other than home	4.2 ≤	135	0.83	0.20–3.63	0.819
	< 4.2	74			
ACS NSQIP surgical risk calculator					
Serious complication (%)	25.6 ≤	88	1.31	0.44–3.95	0.627
	< 25.6	121			
Readmission (%)	16 ≤	107	3.09	0.84–11.33	0.089
	< 16	102			
Death (%)	1.9 ≤	120	1.48	0.44–5.00	0.532
	< 1.9	89			
Discharge to nursing or rehab facility (%)	14 ≤	81	2.50	0.71–8.85	0.154
	< 14	128			
Body composition assessment					
Preoperative visceral fat (cm^3^)	5580 ≤	7	0.12	0.12–7.43	0.355
	< 5580	202			
Preoperative psoas muscle density	38.25 ≤	143	0.51	0.22–1.19	0.117
	< 38.25	66			

Abbreviations: ACS NSQIP, The American College of Surgeons‐National Surgical Quality Improvement Program; ADL, activities of daily living; ASA‐PS, American Society of Anesthesiologists‐physical status; CI, confidence interval; NCD, National Clinical Database; OR, odds ratio.

Univariate logistic regression analysis revealed that comorbid cerebrovascular disease, preoperative antiplatelet medication, prolonged surgery, long hospital stays, high percentages of risk factors calculated by the NCD and ACS risk calculators, high preoperative visceral fat volume, and low preoperative PMD were significantly associated with a poor postoperative course. No significant associations were shown with smoking history, type of primary disease, or surgical procedure. On multivariate analysis, “Predicted incidence of Clavien–Dindo grade IV or higher” of ≥ 9.2% (OR 5.92, 95% CI 1.25–28.00; *p* = 0.025) and “Predicted incidence of postoperative ADL decline” of ≥ 44.8% (OR 4.68, 95% CI 1.15–19.08; *p* = 0.031) were identified as independent predictors of a poor postoperative course.

**FIGURE 2:**
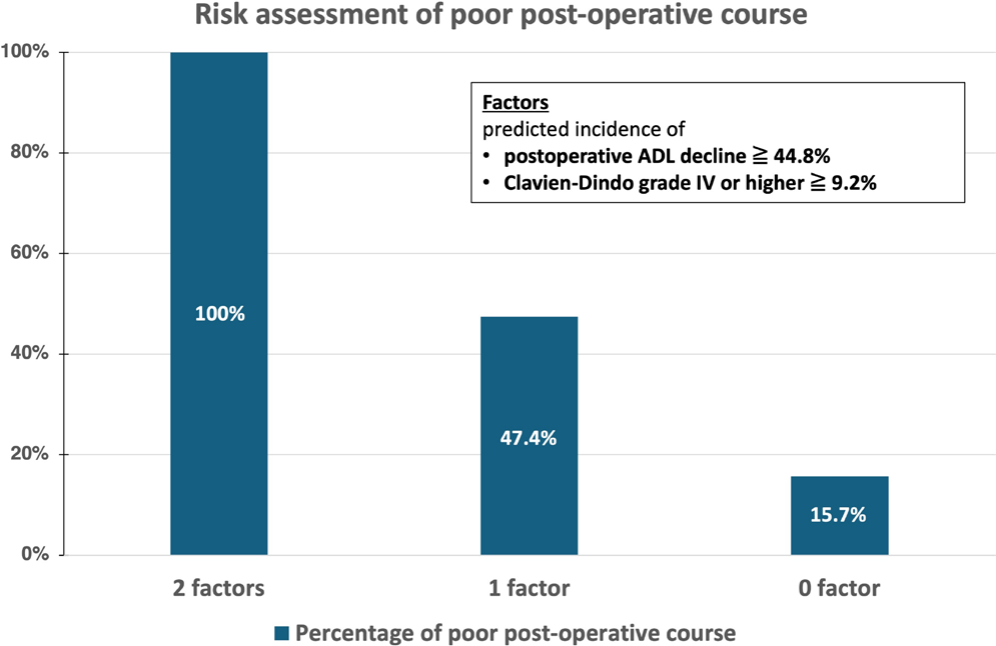
Risk assessment of poor postoperative course. All patients (100%) with two factors had a poor postoperative course. In total, 47.4% of patients with one factor and 15.7% of patients without either of the two factors had a poor postoperative course.

Among the 311 patients, 6 had the above 2 factors, and all six (100%) had a poor postoperative course. Among the 38 patients with one of the above two factors, 18 (47.4%) had a poor postoperative course, and among the 267 patients without either of the above two factors, 42 (15.7%) had a poor postoperative course (Figure 2).

In addition, three items of poor postoperative outcomes were also examined. Among the 311 patients included in this study, postoperative complications classified as Clavien‐Dindo grade IV or higher (A) were observed in 11 cases (3.5%). A total of 37 patients (11.9%) were discharged to a facility other than home (B), while 46 patients (14.8%) experienced a decline in their Barthel Index (C).

Regarding overlapping cases, 7 patients (2.3%) had both severe postoperative complications (A) and were discharged to a facility (B). Similarly, 8 patients (2.6%) had severe complications (A) along with a decline in their Barthel Index (C), while 21 patients (6.8%) experienced both discharge to a facility (B) and a decline in the Barthel Index (C). Notably, 7 patients (2.3%) exhibited all three unfavorable outcomes (A, B, and C) (Table 4).

**TABLE 4:** Number and percentage of patients in the three categories of poor postoperative course.

Category	Cases (*n*)	Proportion (%)
A: Serious postoperative complication (CD Gr IV or V)	11	3.5%
B: Discharge to a facility other than home	37	11.9%
C: Worsening Barthel index	46	14.8%
A ∩ B (Both A and B)	7	2.3%
A ∩ C (Both A and C)	8	2.6%
B ∩ C (Both B and C)	21	6.8%
A ∩ B ∩ C (All three: A, B, and C)	7	2.3%
Total cases	311	100%

Abbreviation: CD, Clavien‐Dindo.

### 3.3 Prediction Model for Poor Postoperative Course Using the NCD Item

A multivariate logistic regression model was used to predict serious complications. The model was based on the NCD risk calculator values ‘Predicted incidence of Clavien–Dindo grade IV or higher’ and ‘Predicted incidence of postoperative ADL decline’, and their interaction terms were used to calculate the probability of a poor postoperative course. The above two items in the risk calculator were binary variables, with ‘0’ assigned for ‘a predicted incidence of Clavien–Dindo grade IV or higher’ if less than 9.2% and ‘1’ for 9.2% or higher. A predicted incidence of postoperative ADL decline was assigned ‘0’ if it was less than 44.8% and ‘1’ if it was 44.8% or higher. The equation for the logistic regression model is as follows:

$$\begin{array}{cc} \mathrm{Log}\;p/1-p= & \text{β0}+{\text{β1}}^{*‘}\text{Predicted incidence of Clavien}–\text{Dindo grade}\;\mathrm{IV}\;\text{or}\;{\text{higher}}^{’}\\ & +{\text{β2}}^{*‘}\text{Predicted incidence of postoperative}\;\mathrm{ADL}\;{\text{decline}}^{’} \end{array}$$

In the above formula, *p* = Pr (poor postoperative course).

In this model, the estimated β0 was −1.702, β1 was 1.787, and β2 was 1.757. “Predicted incidence of Clavien–Dindo grade IV or higher” and “Predicted incidence of postoperative ADL decline” were significantly associated with a poor postoperative course.

### 3.4 Model Evaluation

Model performance was assessed by the AUC of the ROC. The AUC of the ROC calculated with the above two variables using the training data was 0.633. The AUC of the ROC was 0.670 when this model with patient data from the training cohort was fitted to the validation data, indicating acceptable discrimination.

### 3.5 Factors Predicting a Poor Postoperative Course in Elderly PD Patients Over 78 Years of Age

Although initial studies were performed on patients 65 years of age and older, criteria for the indication for surgery in the very elderly are needed more in actual clinical practice. As shown in Table 2, 78 years of age was the cutoff value for a poor postoperative course; therefore, univariate and multivariate analyses were performed in 100 patients over 78 years of age, who are considered very elderly patients requiring special attention.

In the univariate analysis, statistical significance was evident for many of the risk factors calculated by the NCD risk calculator and the ACS risk calculator. On multivariate analysis, not only “Predicted incidence of Clavien–Dindo grade IV or higher” of ≥ 9.2% and “Predicted incidence of postoperative ADL decline compared to preoperative” of ≥ 40.6% but also “Preoperative PMD” < 38.7 (OR 6.59, 95% CI 2.20–19.74; *p* < 0.001) were identified as independent predictors of a poor postoperative course in very elderly patients (Table 5).

**TABLE 5:** Multivariate analysis of risk factors predicting a poor postoperative course in patients over 78 years of age.

		*n*	Multivariate		
			OR	95% CI	*p*
Age	81 ≤	56	2.23	0.77–6.49	0.140
	< 81	44			
NCD risk calculator					
Predicted incidence of Clavien‐Dindo grade IV or higher (%)	9.2 ≤	17	1.74	1.39–7.78	0.040
	< 9.2	83			
Predicted incidence of pancreatic fistula grade C (%)	11.5 ≤	7	12.92	0.69–133.56	0.144
	< 11.5	93			
Predicted incidence of postoperative delirium (%)	16.2 ≤	21	3.17	0.58–17.26	0.183
	< 16.2	79			
Predicted incidence of fall risk (%)	39.8 ≤	12	5.54	0.60–50.89	0.130
	< 39.8	88			
Predicted incidence of physical function decline at 30 days postoperatively (%)	52.5 ≤	18	0.68	0.11–4.23	0.682
	< 52.5	82			
Predicted incidence of postoperative ADL decline compared to preoperative (%)	40.6 ≤	20	4.68	1.58–6.55	0.038
	< 40.6	80			
Predicted incidence of discharge to a place other than home (%)	19.2 ≤	26	1.03	0.24–4.36	0.965
	< 19.2	74			
ACS NSQIP surgical risk calculator					
Serious complication (%)	25.5 ≤	55	1.98	0.26–3.11	0.456
	< 25.5	45			
Readmission (%)	16.0 ≤	71	2.22	0.68–2.94	0.303
	< 16.0	29			
Discharge to nursing or rehab facility (%)	27.0 ≤	31	6.30	0.14–7.42	0.891
	< 27.0	69			
Body composition assessment					
Preoperative subcutaneous fat (cm^3^)	3652.4 ≤	22	2.77	0.28–5.11	0.737
	< 3652.4	78			
Preoperative psoas muscle density	< 38.7	60	6.59	2.20–19.74	< 0.001
	38.7 ≤	40			

Abbreviations: ACS NSQIP, The American College of Surgeons‐National Surgical Quality Improvement Program; ADL, activities of daily living; CI, confidence interval; NCD, National Clinical Database; OR, odds ratio.

## 4 Discussion

Although recent advances in surgical methods and postoperative care have improved the safety of PD, open PD remains highly invasive [13, 14]. As the incidence of pancreatic cancer—the primary indication for PD—has increased [15, 16], and multidisciplinary treatments have improved long‐term survival, the demand for PD is expected to grow [17, 18]. With Japan's rapidly aging population [15], selecting elderly patients for surgery is challenging. Aging is associated with increased comorbidities and declines in fitness and nutritional status, which can lead to longer postoperative bed rest, higher complication rates, increased mortality, and reduced ADL [13, 19]. In this context, surgical indications for elderly patients are still largely based on the surgeon's impression.

Japan's NCD risk calculator is able to calculate predictive values such as perioperative mortality and complication rates using data from over 1.5 million cases annually. However, at this point, there are no clear criteria indicating which of the risk calculator results and at what level the indication for surgery should be reconsidered. Because many predicted incidence values are generated from this risk calculator, it is not possible to determine which one to choose and which one to focus on. Therefore, until now, surgeons have only “somewhat” looked at the risk calculator value as a reference.

In this study, “poor postoperative course” included not only the occurrence of severe postoperative complications but also worsening of the Barthel index compared to the preoperative state and discharge to a facility other than home. In our institution, the discharge criteria include sufficient recovery of physical function, the ability to perform basic ADL independently or with minimal assistance, and no requirement for prolonged inpatient treatment. In this study, we defined “discharge to a facility other than home” as a transfer to a rehabilitation hospital, a long‐term care facility, or another medical institution. As you pointed out, social factors, patient preferences, and family considerations may influence the decision to transfer. However, most patients who required transfer to such facilities exhibited some degree of ADL decline or postoperative complications that necessitated additional care. Therefore, we considered “discharge to a facility other than home” as one of the indicators of poor postoperative recovery in our study. This is because these are the most important outcomes to avoid in surgery for elderly patients. The results of this study showed that the NCD risk calculator indicated that “Predicted incidence of postoperative ADL decline” ≥ 44.8% and “Predicted incidence of Clavien–Dindo Grade IV or higher” ≥ 9.2% were risk factors for poor postoperative outcomes. The ACS risk calculator was also included in the study, but the NCD risk calculator was found to be more noteworthy. If either one of these factors is present in the preoperative risk assessment, approximately half of the patients will have a poor postoperative course, so the indication for surgery or methods to improve this risk factor should be considered. If both factors are present, surgery should be avoided because 100% of patients had a poor postoperative course in our study.

The NCD risk calculator has been used in various clinical studies [20, 21, 22, 23]. However, no previous studies have examined surgical indications for pancreaticoduodenectomy in elderly patients using risk calculator results. The definition of “elderly” varies, with previous studies often using age cutoffs of 70, 75, or 80 years [24, 25, 26]. However, these cutoffs were not statistically derived. In contrast, our study used the Youden index to determine an optimal age cutoff for predicting poor postoperative outcomes. Rather than simply comparing elderly and non‐elderly patients, we analyzed factors influencing postoperative outcomes in both the overall PD cohort and the elderly subgroup. In addition to this, to improve the reliability of the analysis in this study, a validation study was conducted by dividing the cohort into a training cohort and a validation cohort using the internal validation method. This improved the validity and reproducibility of the results compared to analyses in a single cohort.

Furthermore, in our study, of greatest concern was patients over 78 years of age, where a low preoperative PMD was a risk factor for poor postoperative outcomes. There have been various reports on PMDs. Xiao et al. [27] reported that a low preoperative PMD is an independent risk factor for Clavien–Dindo III‐V postoperative complications in rectal cancer surgery in elderly patients. Additionally, a low preoperative PMD has been reported to be a poor prognostic factor in prostate cancer patients [28]. These results may suggest that patients with low preoperative PMD measurements should receive rehabilitation and nutritional therapy early in the preoperative period. Alternatively, avoiding surgery may be an option.

There are several limitations to this study. First, the NCD risk calculator is limited to data collected from Japan and may not be applicable to patients of other nationalities. Second, the safety of open PD depends on the level of surgical skill and the quality of postoperative management at the facility. Although our institution is accredited as a board‐certified training institution by the Japanese Society of Hepato‐Biliary‐Pancreatic Surgery, the results of this study may not be extrapolated to nontraining institutions. Third, Barthel index data were collected retrospectively from medical records. It is not a prospective data collection and therefore, may be partially unreliable. Fourth, in this study, the validation process was based solely on data from a single institution, which limits the generalizability of the findings. To strengthen the external validity of our results, future studies should incorporate extra‐validation through multicenter or diverse cohort data to ensure the robustness and broader applicability of the conclusions. Fifth, there is the possibility of the influence of confounding factors. Although we performed multivariable logistic regression analysis to adjust for confounding, some unmeasured factors, such as frailty, cognitive function, and detailed nutritional status, might have influenced postoperative outcomes. Additionally, social factors, such as the availability of caregivers and the patient's home environment, were not fully accounted for in this study. Future studies with a more comprehensive dataset, including these variables, are warranted to further refine the predictive model. Finally, no set of nutritional or rehabilitation therapies for elderly patients has yet been determined. To confirm the clinical validity of this study, we plan to investigate the effects of preoperative nutritional and rehabilitative supportive care for elderly patients in the future. Further studies are needed for safe open PD in elderly patients and to evaluate postoperative management, using longer observation periods for a larger number of patients.

In conclusion, the NCD risk calculator is useful for predicting poor postoperative complications in patients who are scheduled for open PD. Caution may be necessary in determining the indication for surgery in patients 65 years and older with a high “Predicted incidence of Clavien–Dindo Grade IV or higher” or “Predicted incidence of postoperative ADL decline” in the NCD risk calculator. The results also suggest that extreme caution may be needed in determining the indications for surgery in very elderly patients 78 years and older with low preoperative PMD.

## Author Contributions

**Nana Kimura:** conceptualization, data curation, formal analysis, investigation, methodology, project administration, writing – original draft. **Ayaka Itoh:** conceptualization, methodology, project administration, supervision, writing – original draft. **Ayano Sakai:** data curation, formal analysis, investigation, writing – original draft. **Katsuhisa Hirano:** conceptualization, data curation, formal analysis, investigation, writing – review and editing. **Kenta Yagi:** data curation, formal analysis, investigation, writing – original draft. **Naoya Takeda:** data curation, writing – review and editing. **Kazuto Shibuya:** conceptualization, writing – review and editing. **Isaku Yoshioka:** conceptualization, writing – review and editing. **Kenta Murotani:** formal analysis, investigation, methodology, writing – review and editing. **Tsutomu Fujii:** conceptualization, project administration, writing – review and editing.

## Ethics Statement

The study was reviewed and approved (Ref. No. R2023022) by the Ethics Committee of the University of Toyama and complied with the Strengthening the Reporting of Observational Studies in Epidemiology (STROBE) guidelines. The study conformed to the provisions of the Declaration of Helsinki. Informed consent was obtained from all the participants through an opt‐out form.

## Conflicts of Interest

Author T.F. is an editorial board member of Annals of Gastroenterological Surgery.
